# Machine learning analyses reveal circadian clock features predictive of anxiety among UK biobank participants

**DOI:** 10.1038/s41598-023-49644-7

**Published:** 2023-12-15

**Authors:** Cole Ventresca, Wael Mohamed, William A. Russel, Ahmet Ay, Krista K. Ingram

**Affiliations:** 1 Department of Mathematics, Colgate University, Hamilton, NY USA; 2 Department of Computer Science, Colgate University, Hamilton, NY USA; 3 Department of Psychological and Brain Sciences, Colgate University, Hamilton, NY USA; 4 Department of Biology, Colgate University, Hamilton, NY USA

**Keywords:** Circadian rhythms and sleep, Anxiety

## Abstract

Mood disorders, including depression and anxiety, affect almost one-fifth of the world’s adult population and are becoming increasingly prevalent. Mutations in circadian clock genes have previously been associated with mood disorders both directly and indirectly through alterations in circadian phase, suggesting that the circadian clock influences multiple molecular pathways involved in mood. By targeting previously identified single nucleotide polymorphisms (SNPs) that have been implicated in anxiety and depressive disorders, we use a combination of statistical and machine learning techniques to investigate associations with the generalized anxiety disorder assessment (GAD-7) scores in a UK Biobank sample of 90,882 individuals. As in previous studies, we observed that females exhibited higher GAD-7 scores than males regardless of genotype. Interestingly, we found no significant effects on anxiety from individual circadian gene variants; only circadian genotypes with multiple SNP variants showed significant associations with anxiety. For both sexes, severe anxiety is associated with a 120-fold increase in odds for individuals with *CRY2*_AG(rs1083852)/*ZBTB20*_TT(rs1394593) genotypes and is associated with a near 40-fold reduction in odds for individuals with *PER3-*A_CG(rs228697)/*ZBTB20*_TT(rs1394593) genotypes. We also report several sex-specific associations with anxiety. In females, the *CRY2/ZBTB20* genotype combination showed a > 200-fold increase in odds of anxiety and *PER3/ZBTB20* and *CRY1 /PER3-*A genotype combinations also appeared as female risk factors. In males, *CRY1/PER3*-A and *PER3*-B/*ZBTB20* genotype combinations were associated with anxiety risk. Mediation analysis revealed direct associations of *CRY2/ZBTB20* variant genotypes with moderate anxiety in females and *CRY1/PER3*-A variant genotypes with severe anxiety in males. The association of *CRY1/PER3*-A variant genotypes with severe anxiety in females was partially mediated by extreme evening chronotype. Our results reinforce existing findings that females exhibit stronger anxiety outcomes than males, and provide evidence for circadian gene associations with anxiety, particularly in females. Our analyses only identified significant associations using two-gene combinations, underscoring the importance of combined gene effects on anxiety risk. We describe novel, robust associations between gene combinations involving the *ZBTB20* SNP (rs1394593) and risk of anxiety symptoms in a large population sample. Our findings also support previous findings that the *ZBTB20* SNP is an important factor in mood disorders, including seasonal affective disorder. Our results suggest that reduced expression of this gene significantly modulates the risk of anxiety symptoms through direct influences on mood-related pathways. Together, these observations provide novel links between the circadian clockwork and anxiety symptoms and identify potential molecular pathways through which clock genes may influence anxiety risk.

## Introduction

Mood disorders, including depression and anxiety are becoming increasingly prevalent and affect greater than twenty percent of the world’s adult population^1,2^. Estimates in Europe suggest that anxiety disorders alone affect nearly one-fifth of the population and that current interventions are ill-equipped to treat this complex disorder^3^. Every year, anxiety and depression account for over 60 million disability-adjusted life years (DALYs), threatening work productivity and social relationships, among other economic and personal costs^4^. Efforts to better understand the etiology of mood disorders have uncovered genetic and environmental links between circadian clock-related genes and mood disorders including major depressive disorder (MDD)^5–10^, schizophrenia^11^, bipolar disorder (BD)^7–10^, seasonal affective disorder (SAD)^12^, and anxiety^13,14^. While clinical studies have reinforced the roles of circadian rhythm and clock genes in depressive disorders, less is known about how the circadian clockwork influences anxiety risk^15^.

Circadian rhythms are responsible for the 24-h sleep–wake cycle that revolves around natural and/or artificial light–dark cycles. These rhythms are facilitated by the molecular clock, which regulates over one-third of all transcribed genes responsible for physiological cycles throughout the body through feedback loops in core clock genes and their associated transcription factors^16,17^. While most tissues in the body individually express these genes, the suprachiasmatic nucleus (SCN), located in the hypothalamus, plays a crucial role in synchronizing and modulating expression in response to environmental cues. For example, the SCN integrates light/dark input from the eyes^18,19^ and relays this information to the peripheral clock systems via the secretion of neurotransmitters, neuropeptides, growth factors, and cytokines^20,21^. The transcription factor CLOCK is involved in the core feedback loop, regulating the transcription of *Period* (*PER1*, *PER2*, *PER3*) and *Cryptochrome* (*CRY1* and *CRY2*) gene families to maintain the 24-h circadian rhythm cycle. The CLOCK protein forms a heterodimer with brain and muscle ARNT-like protein-1 (BMAL1), which activates the transcription of additional core clock genes, including *PER* and *CRY*. PER/CRY heterodimers then inhibit CLOCK/BMAL1 activity, forming a negative feedback loop^22^. Mutations in these core clock genes may affect mood directly by altering the downstream physiological pathways involved in mood regulation, or indirectly through the disruption of circadian phase.

Many studies have demonstrated an indirect effect of the circadian clock on mood disorders by linking chronotype or diurnal preferences, differences in sleep–wake timing and activity patterns, with negative affect^13,14,23–27^. These studies support the ‘social jetlag’ hypothesis which posits that alterations in circadian rhythms influence mood by creating a misalignment between an individual’s sleep–wake activity and their social routine^7,26,28–34^. Moreover, genome-wide association studies (GWAS) and candidate gene studies have provided strong evidence that circadian clock genes are associated with diurnal preference, chronotype, and sleep disturbance [^35–39^]. Insights from GWAS studies also suggest overlapping genetic profiles between individuals with altered chronotype and individuals with mood disorders^35,39^. However, GWAS studies on anxiety have not yet identified significant associations between circadian clock genes and risk^40,41^. Core circadian genes may also have direct effects as transcriptional regulators, influencing neurotransmitter signaling in serotonin, dopamine, and glucocorticoid mood pathways^42^. Such links suggest the potential for direct and indirect influences of circadian clock genes on mood disorders, although the molecular mechanisms that facilitate these effects remain poorly understood. In addition, clock genes often affect neurotransmitter signaling and circadian alignment simultaneously, making it difficult to disentangle the indirect and direct effects of clock genes on mood.

Recent studies have demonstrated the involvement of clock genes in mood disorders (Table 1). For example, recent studies have found that the knockdown of *CRY1*^16^, *CRY2*^43^, and *PER3*^44^ clock genes affected anxiety, depression behaviors, and circadian timing. Population-level candidate gene studies^9,13,45^ and mathematical modeling^25^ have also identified clock genes important to mood disorders. Although GWAS studies have struggled to identify genotypes associated with complex mood disorder phenotypes^14^, Ho and colleagues (2018) found that an intronic SNP variant in *ZBTB20* (rs1394593) exhibited a strong association with seasonal affective disorder^46^. This study also found strong association signals for candidate gene targets of ZBTB20, suggesting that *ZBTB20*-regulated pathways have an important role in SAD etiology^46^. In addition, methylome changes in *ZBTB20* have previously been associated with major depressive disorder^47^. However, candidate gene studies have yet to investigate the influence of *ZBTB20* on circadian and mood pathways.

**Table 1:** Summary table depicting the associations of clock genes analyzed in this study with anxiety, depression, and sleep/wake patterns.

	Methods	Significant Clock Genes
**Anxiety**		
Liberman et al.^13^	Mathematical modeling	*PER3*
Liberman et al.^25^	Mathematical modeling	*CRY1, CRY2, PER3-B*
Silva et al.^101^	*PER3* statistical testing	*PER3*
Zafar et al. (2016)	Statistical and ML approaches	*CLOCK, CRY1, PER3-B*
**Depression**		
Davies et al.^47^	Genome-wide methylation analysis	*ZBTB20*
Ho et al.^46^	SAD GWAS	*ZBTB20*
Hua et al.^85^	Candidate gene, case–control	*CRY1*
Kim et al.^24^	Candidate genes, statistical testing	*CLOCK*
Lavebratt et al.^5^	Candidate gene approach	*PER2*
Lavebratt et al.^6^	CRY2* expression and statistical analysis*	*CRY2*
Shi et al.^79^	Molecular/functional	*CLOCK, PER3-A, PER3-B*
Silva et al.^101^	*PER3* statistical approach	*PER3*
Soria et al.^27^	Candidate gene statistical approach	*CRY1*
**Sleep/Wake**		
Archer et al. (2003)	*PER3*, statistical testing	*PER3* (diurnal preference. sleep phase)
Ebisawa et al. (2001)	*PER3*, statistical testing	*PER3* (delayed sleep phase)
Evans et al.^16^	*CRY1*(−/−) mice behavior	*CRY1* (altered phase)
Hida et al.^80^	Candidate genes, statistical testing	*PER3-A* (diurnal preference)
Dashti et al.^35^	GWAS	*PER1* (sleep duration)
Jones et al.^37^	GWAS	*PER2* (SCN enrichment)
Katzenburg et al. (1998)	Candidate genes, statistical testing	*CLOCK* (delayed phase and chronotype)
Overton et al.^38^	Statistical and ML approaches	*CLOCK, CRY1, CRY2, PER3B* (sleep disturbance)
Savalli et al.^43^	*CRY2*(−/−) mice behavior	*CRY2* (diurnal patterns)
Zhang et al.^44^	*PER3*(−/−)	*PER3* (circadian pattern. phase)

A table was created for the genes analyzed in this study, summarizing their previous associations with anxiety, depression, and chronotype, and the methods that each study used.

In this study, we investigate associations of clinical features and SNP variants in core clock genes, including *ZBTB20*, with anxiety (Generalized Anxiety Disorder 7-item scale (GAD-7)) scores in a UK Biobank population of 90,882 individuals, aged 40–69 years old. We explore the effects of single-gene variants and multi-gene variant combinations utilizing a complementary approach of machine learning and statistical methods. Here, we report strong sex-specific associations of circadian gene variants with anxiety symptoms and propose that a variant of *ZBTB20* (rs1394593) influences anxiety risk via amplification or attenuation of anxiety symptoms in combination with other clock variants.

## Methods

### Data sourcing

All data used in the study originates from the UK Biobank, a large prospective study comprised of approximately 500,000 individuals ages 40–69 that were recruited from 2006 to 2010. UK Biobank is a resource for studies investigating the genetic, environmental, and lifestyle determinants of a wide range of diseases present in middle and later life^48^. At the beginning of the initial assessment, written consent was obtained from each study participant^48^. Individuals underwent body measurements, provided lifestyle information and medical history, and donated blood, urine, and saliva samples for genetic and phenotypic investigations^49^. We did not exclude by ethnicity in this study; 94.6% of UK Biobank participants are recorded as ‘White,’ and 5.4% are recorded as ethnic minorities^106^.

### UK biobank features

Supplementary Fig. 1 provides a summary of methods employed in this study. UK Biobank data was translated into a readable form using ukbb_parser^50^. We analyzed the following single-nucleotide polymorphisms (SNPS): *CLOCK* (rs1801260), *PER2* (rs10462023), *PER3* variants *PER3-A* (rs228697), *PER3-B* (rs17031614) and *PER3-C* (rs10462020), *CRY1* (rs2287161), *CRY2* (rs1083852), and *ZBTB20* (rs1394593), which were all collected by UK Biobank researchers. In addition, we examined six behavioral/clinical features: age (ID: 21003), sex (ID:31), chronotype (ID: 1180), household income (ID: 738), substance addiction (ID: 20457), and Townsend Material Deprivation score (ID: 22189). Substance addiction was defined by self-report answers to a survey question asking participants if they had an ongoing addiction or dependence on illicit or recreational drugs, and they were given the answer options “No”, “Yes”, and “Prefer not to answer”. Chronotype was defined by self-report responses where individuals classified themselves as definite morning or evening-types, partial morning or evening-types, or had the option of selecting “I don’t know” or “prefer not to answer”. Household income was also collected through self-report where individuals were asked to report their average household income by selecting one of five pre-specified ranges, also with “I don’t know” or “Prefer not to answer” options. Individuals who selected “I don’t know” or “Prefer not to answer” for any of the covariates of interest were removed from the analysis. Townsend Material Deprivation score was calculated by participant postal code.

Anxiety was assessed using the Generalized Anxiety Disorder 7-item scale^51^. Additional measures related to anxiety are available from the UK Biobank database, including the ICD-10 (International Classification of Disease for anxiety disorder), clinical diagnoses of anxiety, the use of anxiolytic medicine, and self-reported measures of anxiety-related doctor visits and symptoms of anxiety. Although these variables, particularly the clinical diagnoses of and treatments for anxiety, are excellent measures of anxiety disorders, using these measures significantly reduces the sample size for statistical analysis of gene variant associations. Thus, we chose to include the well-supported GAD-7 instrument as the anxiety measure to maximize the sample size for our analyses.

### Feature engineering and selection

To account for the effects of population structure and batch-based genotyping, UK Biobank researchers utilized several stages of quality control [^52,53^]. First, several different SNP-based metrics were used identify and eliminate less reliable genotyping results. If SNPs were missing in multiple batches, then they were removed from analysis [^53^]. SNPs with a minor allele frequency less than 1 percent (MAF < 1%) were removed from analysis [^53^]. Next, researchers focused exclusively on high-quality SNPs to identify poor-quality samples [^53^]. Finally, principal components analysis and relatedness inference were used for sample-based inference. From these quality control steps, UK Biobank researchers identified few SNPs and samples to be removed [^53^]. These researchers performed whole-genome imputation with IMPUTE2 using a diverse reference panel. Imputation information scores were used to assess imputation, and these scores revealed effective imputation for SNPs of varying MAFs [^52^]. In our analysis, any individuals lacking relevant data pertaining to any of our selected features or outcome were removed from the population analyzed; no imputation was performed for these missing values. This left 90,882 UK Biobank participants for analysis.

One-hot encoding was performed to transform categorical variables into numeric values that can be read for machine learning analysis. For categorical variables with *n* categories, *n*-1 new columns were created. One of the categories was considered the reference category and excluded, since it could be inferred from the other columns. With SNP data, for example, two new columns were created for the two less frequent genotypes, and the common genotype column was considered as the reference category (Supplementary Fig. 2). SNP data was also imputed for *CLOCK, PER2, PER3-A*, and *PER3-C*, consistent with the imputation that UK Biobank researchers had already performed on *CRY1, CRY2, PER3-B*, and *ZBTB20* using which were imputed by UK Biobank researchers using the Haplotype Reference Consortium^54^, and UK10K and 1000 Genomes reference panels^55,56^. These 6 clinical features and 8 single-genotype features were examined for associations with anxiety. Also, we investigated gene combinations involving two genotypes and their respective variants to examine pairwise interactions. This generated an additional 8C2*8 two-way features; of eight total genes, any two can exist in a pair and there are eight potential genotypic combinations that paired genes may have, resulting in 224 total genotype combinations.

The GAD-7 is a self-report scale that ranges from 0 to 21. GAD-7 scores have commonly been broken into thresholds where scores ≤ 4 indicate minimal anxiety, 5–9 suggest mild anxiety, 10–14 suggest moderate anxiety, and ≥ 15 indicate severe anxiety. Since a score of 8 is a commonly held cutoff for symptoms of mild anxiety^57,58^, we used this supported cutoff to establish anxiety presence. From here, we adhered to the cutoffs for anxiety severity and used the following thresholds: mild anxiety (8–11), moderate anxiety (12–15), and severe anxiety (≥ 16). Individuals with a GAD-7 score < 8 were used as controls for the data analysis. We split these categories into three different binary outcomes for multiple multivariate logistic regression analyses. Our data was reprocessed four times into four total separate datasets where the data either was or was not one-hot encoded and the outcome variable was either binary or continuous, so that multivariate linear regression, multivariate logistic regression, the Sheirer Ray Hare Test, and mediation analysis could be performed with the correct types of data.

We used a combination of feature selection methods to determine which features to use in our subsequent analyses. We used a combination of multiple ranking-based and subset-based feature selection methods to mitigate the inherent bias of individual feature selection methods, choosing features that were ranked highly by all feature selection algorithms. Chi-square, InfoGain (IG), and ReliefF (ReF) are ranking-based feature selection methods that rank features by their contribution to the disorder outcome. The chi-square method calculates the association between features and anxiety outcomes using the chi-squared score^59^. InfoGain (IG) ranks features based on the amount of entropy each feature explains^60,61^. ReliefF (ReF) scores features based on their value, relative to their nearest-neighbor instance^62^. Joint mutual information (JMI) and minimum redundancy maximum relevance (MRMR) are subset-based feature selection methods. Both of these methods determine subsets in the feature space, and select feature subsets that have the strongest relationships with an outcome and the weakest relationship with other features by evaluating and comparing these two different interactions. Joint mutual information (JMI) selects features for a subset that maximize the cumulative sum of joint mutual information when added to the subset^63^. Minimum redundancy maximum relevance (MRMR) iteratively selects a subset of features that have the most correlation with the class, and the least correlation with other features^64^. Bootstrapping was performed for each feature selection method by running it 50 times and taking features that appeared in the top 50 features at least 70 percent of the time. Then, the results across these five methods were compared using a sum of ranks and the 25 features that performed the best over the five techniques were used in our statistical analysis.

### Statistical analyses

All statistical analyses were performed in R [^65^] and the Scikit-learn library in Python^66^. Our code is available at: https://github.com/cventresca/ukbb_analyzer.

Since there was still high dimensionality in the dataset following feature selection and we observed initial overfitting of our model, we used Variance Inflation Factor (VIF) and Akaike Information Criterion (AIC) as we performed our regressions to identify important features. VIF was used to identify multicollinearity and variables with a VIF score > 10 were excluded from the analysis^67^. We also used AIC, which is a model selection algorithm that uses sequential replacement to identify features with low multicollinearity and strong association with GAD-7^68^. Features that were deemed important by both selection methods, in addition to machine learning feature selection methods described above, were used in subsequent multivariate analyses.

Multivariate linear and logistic regression analyses were performed using the Statsmodels library in R^69^. We performed multivariate linear regression to predict continuous GAD-7 scores with genotypic and clinical independent variables. First, a Durbin-Watson statistic was obtained to check the independence of residuals assumption^70^, and then a scatterplot was constructed to confirm a linear relationship between each independent feature and the collective of independent features with GAD-7 scores. Studentized residuals were plotted against the unstandardized predicted values to check that the assumption of homoscedasticity was met. No outliers, high leverage points, or highly influential points were detected during analysis, and the normality of residuals was confirmed via a histogram with a superimposed normal curve and a P–P Plot. After performing multivariate linear regression, P-value corrections were performed using the Benjamini Hochberg (BH) correction, due to unplanned pairwise comparisons between features^71^. Next, multivariate logistic regression was performed to assess genotypic and clinical predictors for mild, moderate, and severe anxiety outcomes. A linear relationship was confirmed between continuous independent variables and the logit-transformed GAD-7 outcome for all three anxiety classifications, and no outliers, high leverage points, or highly influential points were detected during analysis. Following the analysis, p-values were also adjusted for multivariate logistic regression using the Benjamini Hochberg (BH) procedure^71^.

We sought to analyze the two-way interactions (genotype and sex) of the SNP combinations that appeared in our multivariate analysis using two-way ANOVA. However, the Shapiro–Wilk test of normality showed that our data was not normally distributed^72^. Therefore, to identify sex-specific differences in average GAD-7 scores for two-way gene combinations, we performed the Scheirer Ray Hare Test in R^73^, which instead compares the median GAD-7 scores across groups. Bar plots of these significant combinations revealed that GAD-7 score distributions were similar enough in shape across groups to have their medians compared.

Mediation analysis was performed to examine whether features were directly associated with anxiety or indirectly associated with anxiety through extreme morning or extreme evening chronotype. Our mediation analysis was conducted in the Mediation library in R^74^, and this analysis was completed for SNP combinations that were significant in the multivariate logistic regression analysis and their associated anxiety outcome(s). This analysis was performed two times for each combination: once with extreme morning type as the mediator and once with extreme evening type as the mediator. Clinical variables including addiction, age, income, and Townsend deprivation index scores were used as confounders when determining the mediation effect of chronotype.

## Results

### Risk and protective factors for anxiety

**Figure 1:**
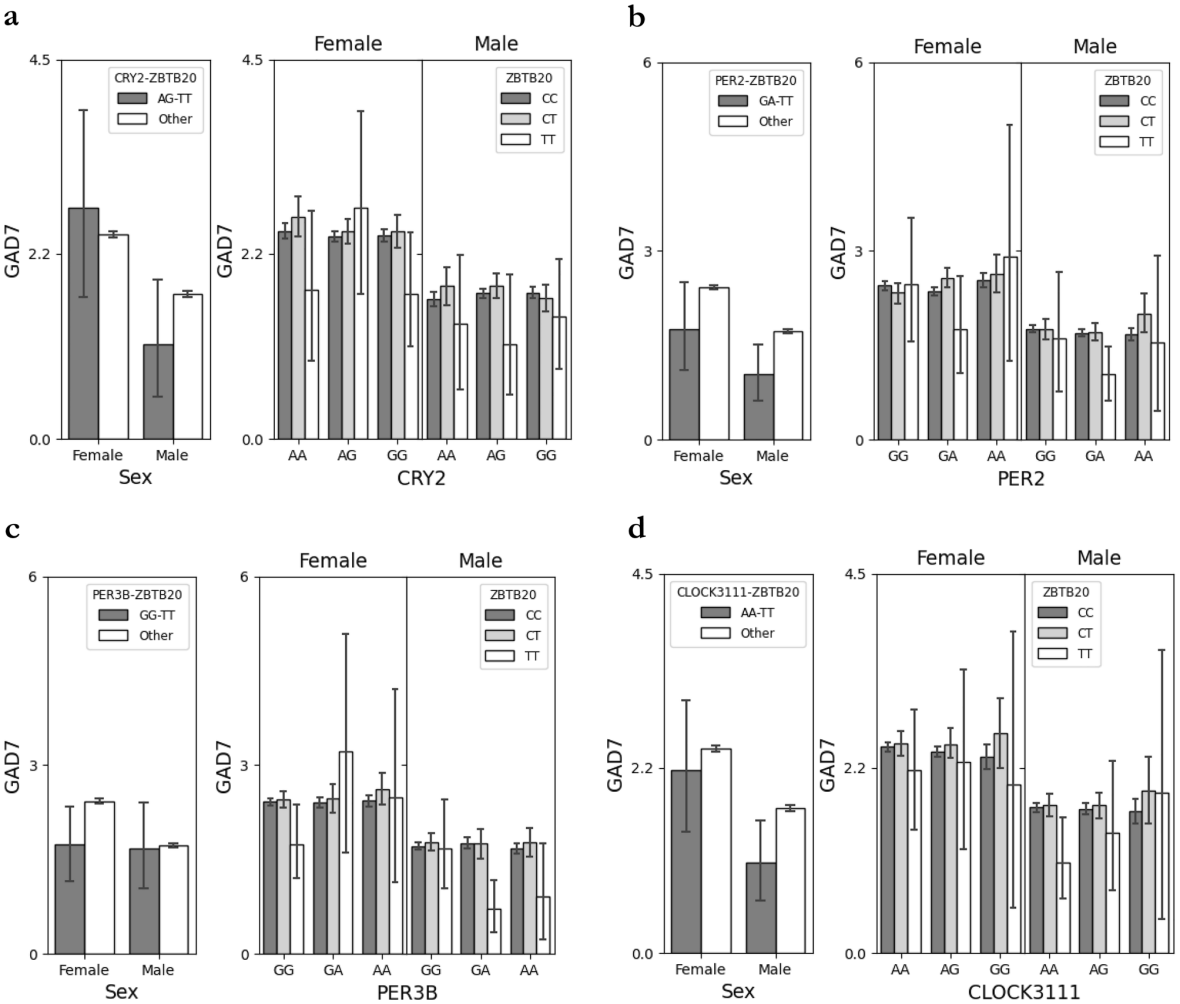
Scheirer-Ray-Hare test reveals the effects of genotype and gender on GAD-7 scores. (**a**) There was an effect of genotype and sex on GAD7 score for CRY2-AG/ZBTB20-TT (Genotype: H_1,60584_ = 4.66, p = 0.031, Gender: H_1,60584_ = 827.94, p < 0.0001, Genotype x Gender: H_1,60584_ = 2.09, p = 0.15). (**b**) There was an effect of genotype and sex on GAD7 score for *CLOCK*-AA/ZBTB20-TT (Genotype: H_1,60584_ = 4.40, p = 0.036, Gender: H_1,60584_ = 828.46, p < 0.0001, Genotype x Gender: H_1,60584_ = 0.51, p = 0.48). (**c**) There was an effect of genotype and sex on GAD7 score for PER2-AG/ZBTB20-TT (Genotype: H_1,60584_ = 10.8, p = 0.0010, Gender: H_1,60584_ = 827.15, p < 0.0001, Genotype x Gender: H_1,60584_ = 0.10, p = 0.75). (**d**) There was an effect of genotype and sex on GAD7 score for PER3-B-GG/ZBTB20-TT (Genotype: H_1,60584_ = 3.42, p = 0.064, Gender: H_1,60584_ = 828.47, p < 0.0001, Genotype x Gender: H_1,60584_ = 1.20, p = 0.27).

Average GAD-7 scores were higher in females than males for several combinations, regardless of genotype (Fig. 1; *CRY2*_AG/*ZBTB20*_TT: H_1,60584_ = 827.94, p < 0.0001; *PER2*_AG/*ZBTB20*_TT: H_1,60584_ = 827.15, p < 0.0001; *PER3-B*_GG/*ZBTB20*_TT: H_1,60584_ = 828.47, p < 0.0001; *CLOCK*_AA/ZBTB20_TT: H_1,60584_ = 828.46, p < 0.0001). Extreme evening type behavior was associated with increased odds of severe anxiety (Table 2; OR 1.4(1.16–1.79) adj. p = 0.0021), mild anxiety (OR 1.1(1.02–1.27) adj. p = 0.028) and was identified as a risk factor by linear regression (estimate 0.01(0.0060–0.015) adj. p = 0.0000036).

**Table 2:** Multivariate linear and logistic regression reveal significant risk and protective factors for anxiety in both sexes.

Feature	Estimate	Mild	Moderate	Severe
Risk				
* CRY2*-AG/*ZBTB20*-TT		13.4	6.06*	120**
Extreme evening type	0.011***	1.14		1.44*
Protective				
* CLOCK*-AA/*ZBTB20*-TT		0.205		
* PER3-*A-CG/*ZBTB20*-TT		0.104		0.024
* PER2*-AG/*ZBTB20*-TT	− 0.029			
Extreme morning type	− 0.005***	0.868**	0.765***	
Moderate morning type			0.852*	

Multivariate linear and logistic regression were performed for genotypic and clinical features in association with anxiety. Values under the estimate column from multivariate linear regression provide constant estimates, where values > 0 indicate a risk effect and values < 0 indicate a protective effect. Values in mild, moderate, and severe columns indicate odds ratios provided by multivariate logistic regression, where values > 1 indicate a risk effect and values < 1 indicate a protective effect (*p < 0.01, **p < 0.001, ***p < 0.0001).

For both sexes, *CRY2*_AG/*ZBTB20*_TT was associated with a 120-fold increase in odds of severe anxiety (OR 120(10.1–1420) adj. p = 0.00029), a sixfold increase in odds of moderate anxiety (OR 6.1(1.99–18.50) adj. p = 0.0020), and a 13-fold increase in odds of mild anxiety (OR 13.4(2.02–89.20) adj. p = 0.011). *PER3*-A_CG/*ZBTB20*_TT was associated with a near 40-fold reduction in odds of severe anxiety (OR 0.02(0.0011–0.57) adj. p = 0.034), and a ten-fold reduction in odds of mild anxiety (OR 0.1(0.013–0.87) adj. p = 0.045). *CLOCK*_AA/*ZBTB2*0_TT was associated with a five-fold reduction in odds of mild anxiety (OR 0.2(0.05–0.80) adj. p = 0.09). *PER2*_AG/*ZBTB20*_TT was revealed as a protective factor by linear regression (estimate − 0.03(− 0.054 to − 0.0040) adj. p = 0.021).

### Sex-specific risk and protective factors for anxiety in males and females

In females, being an extreme evening type was associated with 1.6 times the odds of severe anxiety (Table 3; OR 1.6(1.22–2.08) adj. p = 0.0011), and eveningness appeared as a risk factor in multivariate linear regression (estimate 0.01(0.005–0.018) adj. p = 0.00088). Being an extreme morning type was associated with reduced odds of moderate anxiety (OR 0.8(0.68–0.86) adj. p = 0.0000045), mild anxiety (OR 0.8(0.77–0.92) adj. p = 0.00040) and was also identified as a protective factor by multivariate linear regression (estimate − 0.006(− 0.010 to − 0.0030) adj. p = 0.0012).

**Table 3:** Multivariate linear and logistic regression reveal risk and protective factors for anxiety in females.

Female feature	Estimate	Mild	Moderate	Severe
Risk				
CRY1-GG/PER3-A-GG				2.53*
CRY2-AG/ZBTB20-TT	0.060	43.4	219.0*	
CRY2-GG/ZBTB20-TT			24.6	
Extreme evening type	0.011**			1.60*
Protective				
PER3-A-CC/ZBTB20-TT			0.023	
PER3-A-CG/ZBTB20-TT		0.037	0.016	
Extreme morning type	− 0.006*	0.844**	0.723***	

Multivariate linear and logistic regression were performed for genotypic and clinical features in association with anxiety. Values under the estimate column from multivariate linear regression provide constant estimates, where values > 0 indicate a risk effect and values < 0 indicate a protective effect. Values in mild, moderate, and severe columns indicate odds ratios provided by multivariate logistic regression, where values > 1 indicate a risk effect and values < 1 indicate a protective effect (*p < 0.01, **p < 0.001, ***p < 0.0001).

In females, *CRY2*_AG/*ZBTB20*_TT was associated with an > 200-fold increase in odds of moderate anxiety (OR 219(9.02–5.32E + 03) adj. p = 0.0016), and a > 40 times increase in odds of mild anxiety (OR 43.4(2.34–807) adj. p = 0.017). *CRY2*_AG/*ZBTB20*_TT also appeared as a risk factor in multivariate linear regression (estimate 0.060(0.012–0.109) adj. p = 0.017). *CRY2*_GG/*ZBTB20*_TT was associated with ~ 25-fold increased odds of moderate anxiety (OR 24.6(1.28–471) adj. p = 0.039). *CRY1*_GG/*PER3*-A_GG was associated with a 2.5-fold increase in odds of severe anxiety (OR 2.5(1.32–4.86) adj. p = 0.0093.

Also, logistic regression revealed that *PER3*-A_CG/*ZBTB20*_TT was associated with a near five-fold reduction in odds of moderate anxiety (OR 0.02(0.00057–0.47) adj. p = 0.02), and a greater than 20-fold decrease in odds of mild anxiety in females (OR 0.04 (95% CI) adj. p = 0.049). *PER3*-A_CC/*ZBTB20*_TT was associated with a near five-fold decrease in odds of moderate anxiety (OR 0.02(0.0011–0.49) adj. p = 0.02).

For males, multivariate linear regression identified extreme evening type as a risk factor (Table 4; estimate 0.01(0.0040–0.016) adj. p = 0.0016). Also, being an extreme morning type was identified as a protective factor for mild anxiety by logistic regression (OR 0.9(0.75–0.98) adj. p = 0.028) and was revealed as protective by linear regression in males (estimate − 0.004(− 0.0080–0.000) adj. p = 0.033). In addition, *CRY2*_AG/*ZBTB20*_TT was associated with a ~ 20-fold increase in odds of severe anxiety (OR 22.5(2.72–186) adj. p = 0.0078). *CRY1*_CC/*PER3*-A_GG was associated with nearly three times the odds of severe anxiety (OR 2.8(1.22–6.49) adj. p = 0.028). *PER3*-B_GG/*ZBTB20*_TT was associated with a ~ sevenfold increase in odds of mild anxiety (OR 7.3(1.47–36.60) adj. p = 0.024).

**Table 4:** Multivariate linear and logistic regression reveal significant risk and protective factors for anxiety in males.

Male feature	Estimate	Mild	Moderate	Severe
Risk				
CRY1-CC/PER3-A-GG				2.81
CRY2-AG/ZBTB20-TT				22.5*
PER3-B-GG/ZBTB20-TT		7.33		
Extreme evening type	0.010*			
Protective				
Extreme morning type	− 0.004	0.853		

Multivariate linear and logistic regression were performed for genotypic and clinical features in association with anxiety. Values under the estimate column from multivariate linear regression provide constant estimates, where values > 0 indicate a risk effect and values < 0 indicate a protective effect. Values in mild, moderate, and severe columns indicate odds ratios provided by multivariate logistic regression, where values > 1 indicate a risk effect and values < 1 indicate a protective effect (*p < 0.01, **p < 0.001, ***p < 0.0001).

### Mediation analyses reveal direct and mediated effects on anxiety symptoms

**Figure 2:**
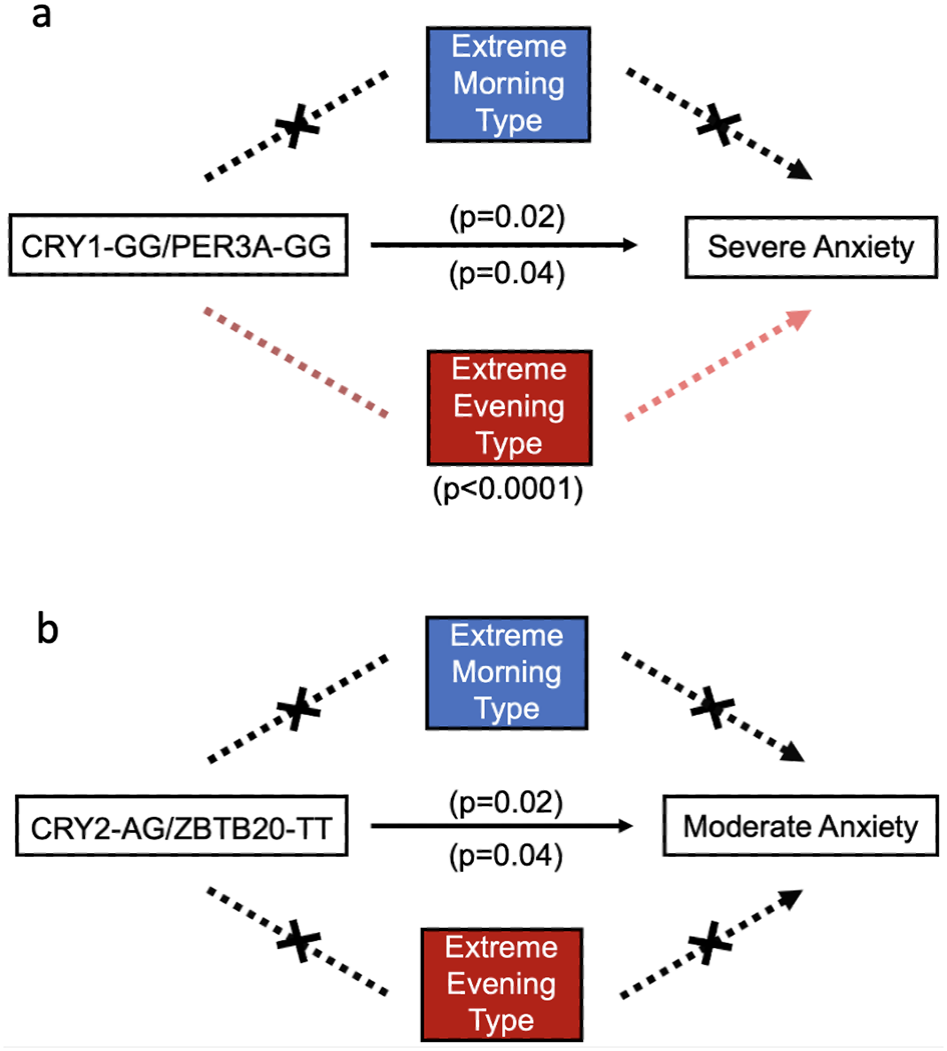
Mediation analysis reveals a combination directly associated with anxiety and a combination whose association is partially mediated by chronotype in females. Mediation analysis was performed in females for mild, moderate, and severe anxiety classifications with extreme morning type and extreme evening type entered as potential mediators. The black line from genotypic combination to outcome indicates a direct effect. Red dotted lines indicate a mediation effect through extreme evening type, and black dotted lines that are crossed indicate no mediation effect. (**a**) A direct effect was observed between CRY1_GG/PER3-A_GG and severe anxiety with extreme morning type as the mediator (p = 0.02), and a partial mediation effect was observed with extreme evening type as the mediator (direct p = 0.04; indirect p = 2E-16). (**b**) A direct effect between CRY2_AG/ZBTB20_TT with moderate anxiety was observed (direct morning p = 0.02; direct evening p = 0.04).

**Figure 3:**
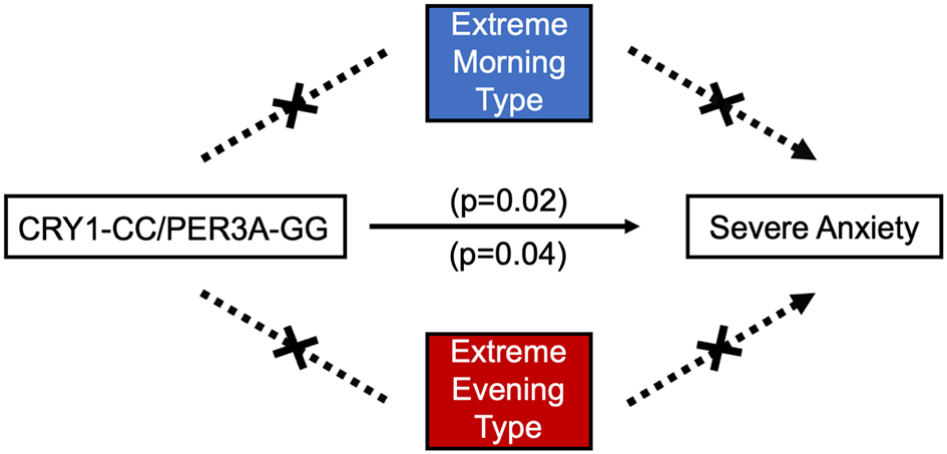
Mediation analysis reveals CRY1_CC/PER3-A_GG is directly associated with severe anxiety in males. Mediation analysis was performed in males for mild, moderate, and severe anxiety classifications with extreme morning type and extreme evening type entered as potential mediators. The black line from genotypic combination to outcome indicates a direct effect. Red dotted lines indicate a mediation effect through extreme evening type, and black dotted lines that are crossed indicate no mediation effect.

In females, *CRY2*_AG/*ZBTB20*_TT exhibited a direct association with moderate anxiety (Fig. 2b; morning type estimate = 0.047(0.0054–0.12) p = 0.020; evening type estimate = 0.054(0.0063–0.15) p = 0.020). *CRY1*_GG/*PER3*-A_GG displayed two associations with severe anxiety: a direct association (Fig. 2a; morning type estimate = 0.017(0.0034–0.040) p = 0.02; evening type estimate = 0.018(0.0040–0.040) p = 2.0E−16), and one mediated by extreme evening type (Fig. 3a; evening type estimate = − 0.00056(− 0.0011–0.00) p = 2.0E-16). In males, *CRY1*_CC/*PER3*-A_GG exhibited a direct association with severe anxiety (Fig. 3; morning type estimate = 0.012(0.0011–0.040) p = 0.02; evening type estimate = 0.017(0.00098–0.050) p = 0.04).

### Decision trees summarize genotypic associations with anxiety symptoms

**Figure 4:**
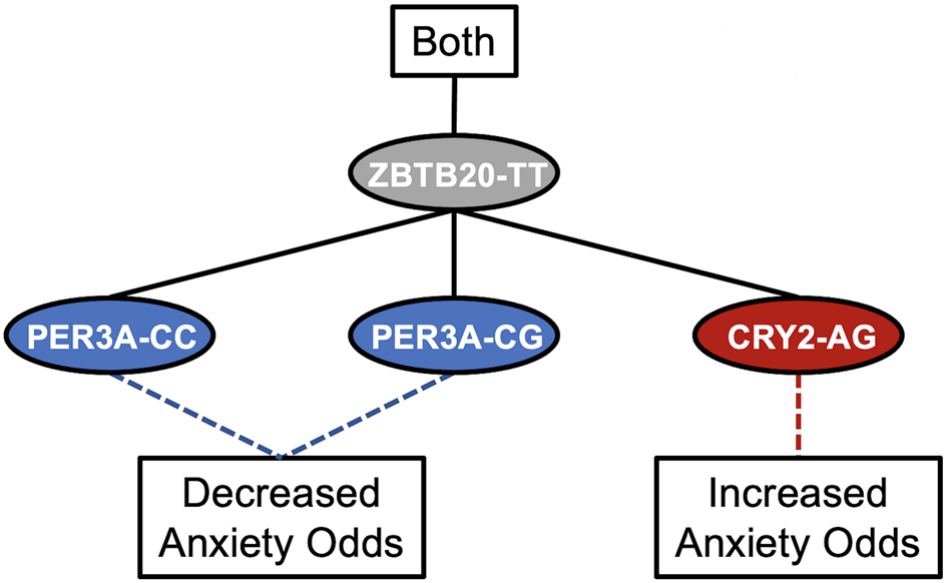
Decision tree summarizes multivariate and mediation analysis findings for genotypes associated with anxiety in both sexes. A decision tree was constructed to visualize associations between clock genes and anxiety that appeared in the overall dataset. Red and blue ovals represent genotypes belonging to risk and protective combinations, respectively, while gray ovals represent genotypes belonging to both risk and protective combinations. Dashed lines represent associations with anxiety, supported by multivariate regression, while solid lines represent effects found by mediation analysis.

**Figure 5:**
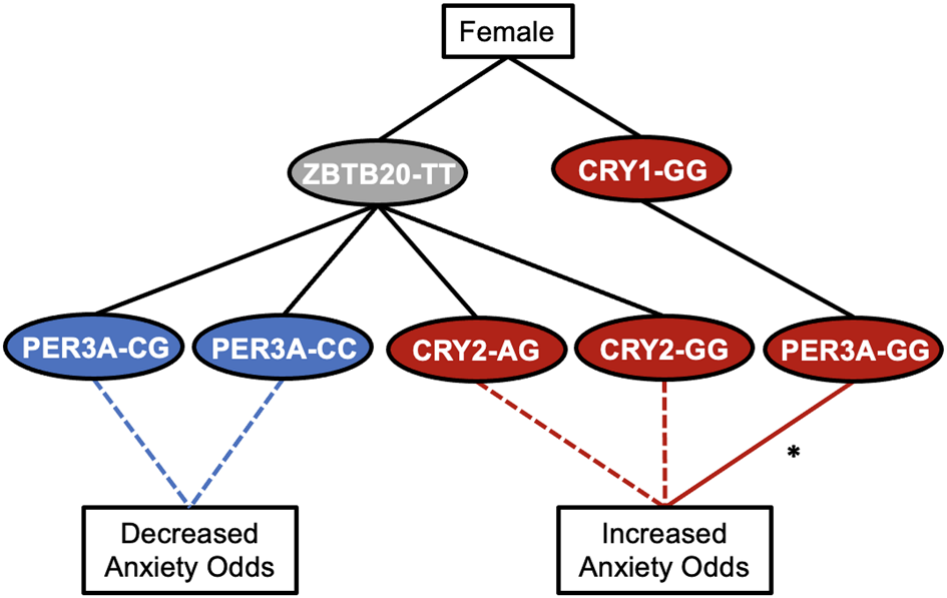
Decision tree summarizes multivariate and mediation analysis findings for genotypes associated with anxiety in females. A decision tree was constructed to visualize associations between clock genes and anxiety that appeared in females. Red and blue ovals represent genotypes belonging to risk and protective combinations, respectively, while gray ovals represent genotypes belonging to both risk and protective combinations. Dashed lines represent associations with anxiety, supported by multivariate regression, while solid lines represent effects found by mediation analysis (*Exhibited partial mediation by extreme evening type).

**Figure 6:**
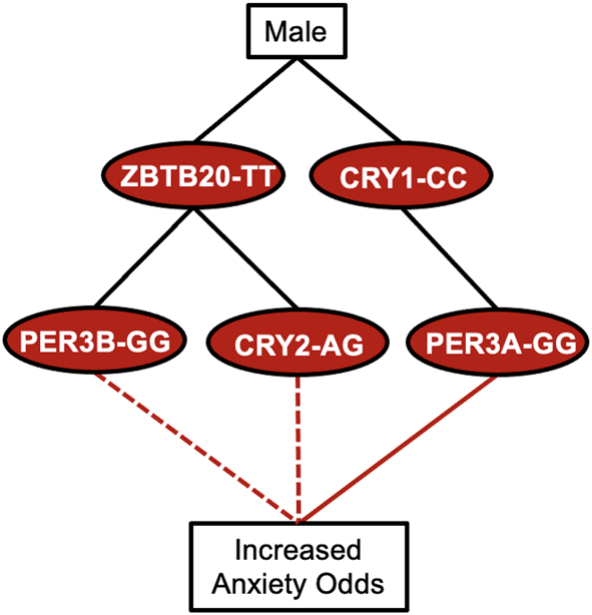
Decision tree summarizes multivariate and mediation analysis findings for genotypes associated with anxiety in males. A decision tree was constructed to visualize associations between clock genes and anxiety that appeared in males. Red ovals represent genotypes belonging to risk combinations. Dashed lines represent associations with anxiety, supported by multivariate regression, while solid lines represent effects found by mediation analysis.

For both sexes, *ZBTB20* variants occurred in a risk combinations with *CRY2*_AG and protective combinations with *PER*3-A_CG (Fig. 4). We constructed association networks to provide a visual summary of our analysis. For females, the *CRY1*_GG/*PER3*-A_GG combination was associated with severe anxiety both directly and indirectly through extreme evening type (Fig. 5). *ZBTB20* was associated with increased anxiety risk in combinations with *CRY2*_AG and *CRY2*_GG and was associated with decreased anxiety risk in combinations with *PER3*-A_CG and *PER3*-A_CC (Fig. 4). In males, the *CRY1*_CC/*PER3*-A_GG combination was directly associated with severe anxiety, and *ZBTB20* variants occurred in risk combinations with *CRY2*_AG and *PER3*-B_GG (Fig. 6).

## Discussion

There is a growing body of evidence supporting the roles of clock gene variants and circadian disruption in anxiety. However, many of these studies have utilized GWAS and PheWAS approaches making it difficult to detect synergistic effects between genotypes or to explore whether significant genotypes are directly or indirectly associated with anxiety. Novel machine learning approaches have shown promise in illuminating these synergistic effects and proposing potential mechanisms of clock pathways that may influence anxiety^14^ and sleep disturbance^38^. Utilizing similar machine learning approaches to analyze clock gene associations with anxiety in a large UK Biobank dataset, we report three main findings: (1) Clock genotype combinations including ZBTB20 variants exhibit combination-specific effects on anxiety, (2) Clock variant combinations associated with anxiety tend to display sex-specific effects, and (3) Circadian-related variants linked to anxiety risk have both direct (chronotype-independent) influences and indirect (chronotype-mediated) influences on anxiety symptoms.

### Genotype combinations with ZBTB20 exhibit diverse associations with anxiety

In this study, we did not observe any single-gene associations with anxiety; only genotype combinations were identified as significant predictors for GAD-7 outcomes. *ZBTB20*_TT was present in nearly every risk combination—with *CRY2* and *PER3*-B and protective combination—with *CLOCK* and *PER3*-A. These findings reinforce results from previous GWAS and target gene studies and suggest that *ZBTB20*_TT may have an important regulatory effect on clock genes involved in anxiety. ZBTB20 is a zinc finger transcriptional repressor protein that is abundant in the hippocampus and is known to have an important role in hippocampal development^75,76^. Previous research from Ho et al.^46^ revealed that the minor T-allele of *ZBTB20* was associated with lower *ZBTB20* mRNA expression and an increased risk for seasonal affective disorder (SAD)^46^. These authors also found that 32 genes associated with SAD were enriched when ZBTB20 levels were reduced^46^ suggesting that ZBTB20 plays an important role in the regulation of clock gene expression. Indeed, other studies have found that ZBTB20 loss is associated with impaired circadian rhythms^77^, and that epigenetic changes inhibiting *ZBTB20* expression are associated with MDD^47^.

Our findings provide further support for ZBTB20 as a regulator for circadian clock genes and demonstrate that genotype combinations that include *ZBTB20* variants can exhibit sex-specific outcomes on anxiety symptoms. In previous studies, the A-allele of *CRY2* has been associated with chronicity patterns characteristic of depressive symptoms^78^, and *CRY2*_AG has appeared in a risk combination for anxiety^14^. Decreases in *CRY2* mRNA have previously been observed in depressed bipolar patients^6^, suggesting that ZBTB20 could act as a repressor of another gene that represses *CRY2* transcription. Therefore, reductions in ZBTB20 could indirectly contribute to lower *CRY2* expression.

This trend was also observed in females who exhibited protective *PER3*-A combinations with *ZBTB20*_TT (*PER3*-A_CC/*ZBTB20*_TT and *PER3*-A_CG/*ZBTB20*_TT). The *PER3*-A G-allele and GG genotype have previously been associated with MDD^25,79^, anxiety^13^, and eveningness^25,80^. Previous mathematical modeling insights suggest that greater PER3 stability contributes to slight increases in period and large reductions in clock amplitude, contributing to circadian misalignment^25^. Thus, ZBTB20 could serve as a repressor for the transcription of *PER3*.

In males, *PER3*-B_GG/*ZBTB20*_TT was associated with increased odds of mild anxiety. However, the A-allele of *PER3*-B has previously been the allele associated with increased odds of MDD and anxiety^14,79^, suggesting a potential combination-specific effect. Indeed, increases in *PER3*-B expression have been shown to associate with circadian disruption^19,81^. Loss of ZBTB20 repression activity may lead to increased *PER3-B* expression and greater circadian disruption, influencing the likelihood of anxiety symptoms.

Finally, we observed that the *CLOCK*_AA/*ZBTB20*_TT and *PER2*_AG/*ZBTB20*_TT genotype combinations were protective factors for both sexes. The C-allele of CLOCK has been associated with MDD^79^, seasonal depression^24^, and evening chronotype^82^, supporting a protective association for *CLOCK*_AA/*ZBTB20*_TT in both sexes. The G-allele of *PER2* has previously been associated with depression vulnerability^5^, so this protective effect may function through alterations in *PER2* expression. As the transcription of *PER2* is finely tuned in response to environmental light^83^ and increased PER2 stabilization leads to circadian disruption in mice^84^, ZBTB20 may act to inhibit a repressor of the *PER2* gene.

### Genotype combinations exhibit sex-specific associations with anxiety

Our main findings in the regression analyses provide support for sex-specific associations of circadian genotypes with anxiety. *CRY1*_GG/*PER3*-A_GG, *CRY2*_GG/*ZBTB20*_TT, and *CRY2*_AG/*ZBTB20*_TT were risk factors that showed stronger associations in females. Also, *PER3*-A_CG/*ZBTB2*0_TT and *PER3*-A_CC/*ZBTB20*_TT were protective for mild and moderate anxiety in females. In males, *CRY1*_CC/*PER*3-A_GG was a risk factor for severe anxiety and *PER3*-B_GG/*ZBTB20*_TT was a risk factor for mild anxiety. Sex-specific associations with circadian genes have previously been observed for anxiety^14^ and major depressive disorder (MDD)^79^.

Sex-specific associations of *CRY2/ZBTB20* and *PER3-A/ZBTB20* with anxiety in females suggest that these combinations are involved in sex-specific pathways. We observed that CRY2 combinations (*CRY2*_GG/*ZBTB20*_TT and *CRY2*_AG/*ZBTB20*_TT) showed significantly stronger associations with anxiety in females than in males. Because *CRY2*_AG/*ZBTB20*_TT also appeared as a risk factor in males, this combination may exert effects through a shared pathway in both sexes. The sex-specific association of *PER3*-A in females is supported by a previous association with MDD^79^. We also observed that *CRY1*_GG/*PER*3-A_GG was associated with severe anxiety in females. Since the C-allele of *CRY1* is the risk allele associated with depression^25,27,85^, and the G-allele of *PER3*-A is the risk allele associated with MDD^25,79^, anxiety^13^, and eveningness^25,80^, these findings provide further support for the sex-specific involvement of *PER3*-A in female anxiety risk.

In males, *CRY1*_CC/*PER3*-A_GG was a risk factor for severe anxiety. Since this combination includes the risk alleles for both genes, this combination could indicate that both genotypes affect anxiety symptoms independently, or in a combination-specific manner. Also, *PER3*-B_GG/*ZBTB20*_TT appeared as a risk factor for mild anxiety in males and this genotype combination does not appear to be a risk factor for females.

There are multiple pathways by which clock gene variants may exert sex-specific effects. Glucocorticoid regulation may be a potential sex-dependent pathway by which these genotypes modulate one’s odds of anxiety^86^. The glucocorticoid pathway has previously been implicated in mood disorders^87^ and clock gene pathways modulate the release of and sensitivity to glucocorticoids^88^. In addition, *PER3*-A and *PER3*-B have been identified in several associations with the sleep–wake cycle and diurnal preference^13,38,80^, which are hypothesized to alter mood through the regulation of serotonin^89–91^. As the function of the 5-HT serotonin system is intertwined with the circadian system^92,93^, and this system affects mood^94^, serotonin regulation has been implicated as a pathway by which circadian disruption can lead to effects on mood^82,89,95,96^. Furthermore, anxiety symptoms have been shown to associate closely with shifts in serotonin activity^97,98^, and sex differences have been observed in serotonergic transmission^99,100^. Therefore, circadian disruptions due to *PER3*-A and *PER3*-B variants could affect mood in a sex-specific manner through alterations in 5-HT signaling. These suggested pathways are supported by previous GWAS studies on anxiety, which have identified other genes known to function in neurotransmitter signaling pathways^40,41^. However, previous GWAS on anxiety have not yet identified associations of clock genes with anxiety.

### Effects on anxiety may be direct or mediated through chronotype

We observed that *CRY2*_AG/*ZBTB2*0_TT was directly associated with moderate anxiety in females, suggesting this genotypic combination exerts direct effects on mood in females. Previously, Zafar et al.^14^ also found that *CRY2*_AG was directly associated with anxiety symptoms^14^. The results of the current study suggest that decreases in the transcriptional repression of CRY2 by ZBTB20 may lead to greater transcription of *CRY2*, which exerts direct effects on anxiety through mood-related pathways.

For both sexes, our multivariate analyses revealed that extreme evening type behavior was associated with an increased risk of anxiety, while extreme morning type was protective against anxiety. Our findings are supported by large-scale GWAS studies that identified clock genes involved in the core feedback loop to be associated with alterations in sleep/wake timing [^35,37^]. Silva and colleagues (2020) suggested that genotypic variants associated with shifts in chronotype may indirectly affect one’s odds of anxiety through the development of symptoms characteristic of various mood disorders^101^. Interestingly, we found that the association between *CRY1*_GG/*PER3*-A_GG and severe anxiety in females was partially mediated by extreme evening type behavior. *CRY* genes activate the circadian loop and function in the retina as light-independent inhibitors of CLOCK/BMAL heterodimers^102,103^, suggesting their role in circadian rhythm maintenance. As stated above, this PER3-A variant (rs228697) has previously been associated with evening type behavior^13,25^, and a significantly higher risk of anxiety^13^. Altogether, these findings suggest that the modulation of *CRY1* expression may lead to alterations in sex-specific mood pathways and diurnal preference pathways in ways that are conducive to anxiety in females. Interestingly, the anxiety risk associated with the co-occurrence of *CRY1*_GG with *PER3*-A_GG in females is similar in magnitude to the male-specific association for *CRY1*_CC/ *PER3*-A_GG genotypes, suggesting that *CRY1* homozygotes, in the presence of *PER3*-A_GG, may affect anxiety via distinct sex-specific mechanisms.

### Limitations

Previous studies have suggested that the UK Biobank population may have a “healthy volunteer” selection bias because only 5% of recruited individuals responded to the invitation. Thus, the Biobank cohort study may not be representative of the UK population^104,105^. For example, UK Biobank study participants were less likely to be socioeconomically deprived, obese, smoke, drink alcohol on a daily basis, and have self-reported health conditions^106^. To counteract the healthy volunteer bias present in the UK Biobank, we controlled for issues of economic status and sex to maximize the generalizability of our results. The UK population and, accordingly, the UK Biobank participants, are predominantly of European Caucasian descent with nearly 95% of the database identifying as ‘White’. Although we did not exclude by ethnicity, our results may not be generalizable to populations of non-Caucasian descent given the small representation of ethnic minorities in the analyses. In addition, the UK Biobank offers additional measures of anxiety, including clinical diagnoses, that could be used to test for associations of clock genes with anxiety. However, these measures offered smaller sample sizes relative to the GAD-7 instrument. Thus, we chose to utilize the well-supported GAD-7 instrument to maximize the power of our analyses. Finally, to minimize computational requirements, we selected circadian gene variants that had been linked to chronotype and/or mood disorders in previous studies and did not study all possible circadian-related variants. Therefore, this study may be missing important circadian features that influence anxiety.

## Conclusions

In this study, we report sex-dependent, combination-specific, and indirect and direct effects of circadian genotypes on anxiety. *ZBTB20* was a feature in several risk and protective genotypic combinations, occurring with *CRY2* and *PER3*-B in risk combinations, and with *CLOCK* and *PER3*-A in protective combinations. Several additional clock-related genes were involved in sex-specific associations with anxiety and these polymorphisms likely influence pathways involved in glucocorticoid and serotonin regulation. Together, these observations reinforce previous GWAS insights into the associations of *ZBTB20* with mood pathways and suggest that ZBTB20 may have a critical regulatory role, both as a repressor and indirect activator, in the transcription of clock genes. In females, we found that the *CRY2*_AG/*ZBTB2*0_TT genotype, our strongest predictor of anxiety, was directly associated with anxiety. The *CRY1*_GG/*PER3*-A_GG genotype in females exhibited effects partially mediated by extreme evening-type behavior, suggesting that circadian effects on anxiety can be both direct and/or mediated by chronotype.

### Supplementary Information

Supplementary Information.

## Data Availability

UK Biobank data are available upon an application process, accessed at: https://www.ukbiobank.ac.uk/enable-your-research.
