# Supplementary material for: Machine learning analyses reveal circadian clock features predictive of anxiety among UK biobank participants
Source: Sci Rep. 2023 Dec 15;13:22304. doi: 10.1038/s41598-023-49644-7 (PMC10724169; doi:10.1038/s41598-023-49644-7)
Supplement: Supplementary file 1 — Supplementary Information. [file 41598_2023_49644_MOESM1_ESM.docx]

**
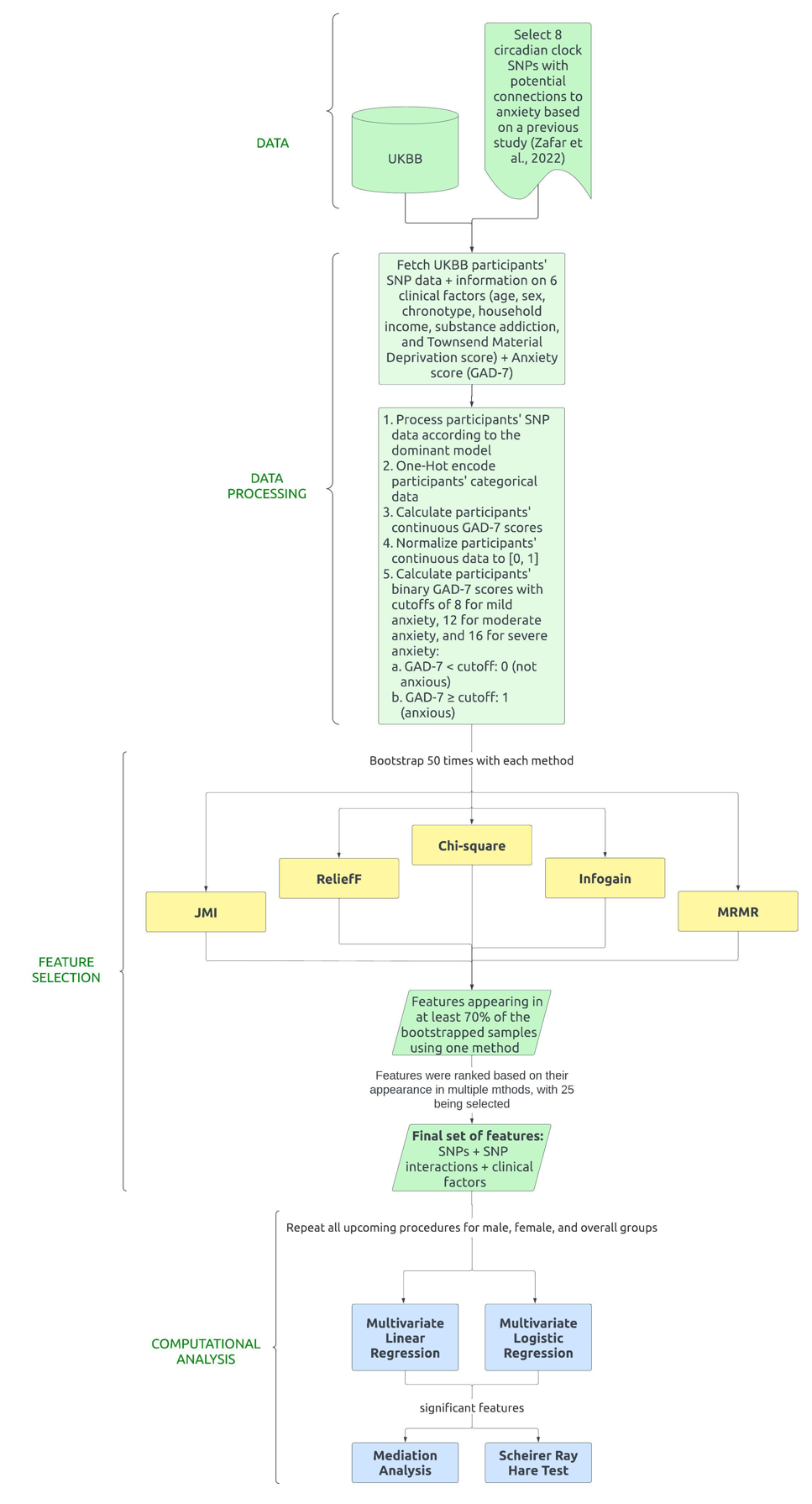
Supplementary Figure 1.** Flow chart of machine learning and statistical analysis methods.

**Supplementary Figure 2.** Depiction of one-hot encoding scheme used for categorical variables, with CRY2 example. Following one-hot encoding (OHE) of our CRY2 SNP data, one column is created for the AG genotype and one column is created for the GG genotype. The common genotype (AA) is considered the reference category and discarded since it can be inferred from the other two columns.

**
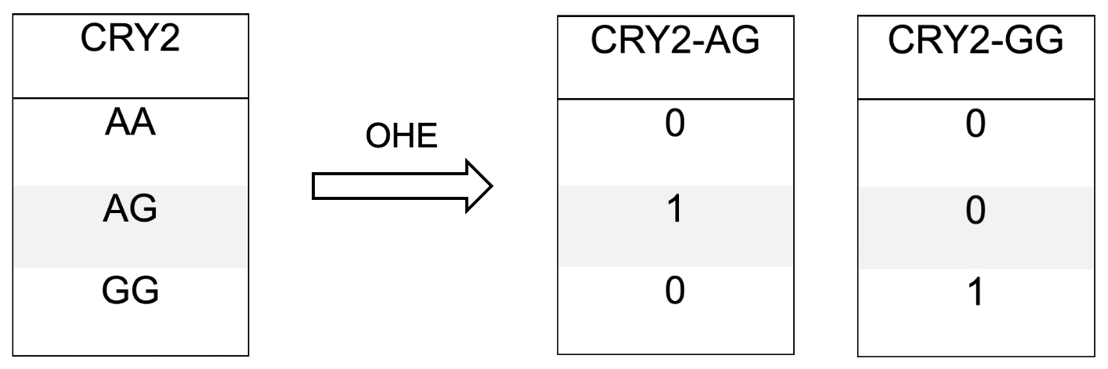
**
